# Which Consumers Change Their Food Choices in Response to Carbon Footprint Labels? The Role of Political Ideology and Other Socio-Demographic Factors

**DOI:** 10.3390/nu17081321

**Published:** 2025-04-10

**Authors:** Julia Diana Lenk, Pierre Chandon, Shemal Doshi

**Affiliations:** 1 Institute of Marketing, University of Hamburg Business School, Moorweidenstraße 18, 20148 Hamburg, Germany; julia.diana.lenk@uni-hamburg.de; 2 Marketing Department, INSEAD, Boulevard de Constance, 77300 Fontainebleau, France; shemal.doshi@insead.edu

**Keywords:** eco-label, carbon labeling, sustainable food choices, political ideology, socio-demographics, marketing

## Abstract

**Background/Objectives**: The effectiveness of eco-labels in encouraging more sustainable food choices varies across studies. We investigate whether consumers’ characteristics may explain this heterogeneity in the context of carbon footprint labeling by studying the moderating role of sociodemographic factors (age, gender, ethnicity, occupation), socioeconomic status (education and subjective socioeconomic position), place of residence (rural to urban), and political ideology. **Methods**: We manipulated the proportion of carbon-labeled products in two incentive-compatible and pre-registered choice experiments. The first (n = 715) asked consumers to shop for instant meal products in an online grocery store containing a food category’s complete product assortment. The second (n = 1233) forced consumers to make tradeoffs between product preferences and carbon emissions in two consecutive food choices for cut fruit products, one without and another with carbon labels. To capture potential lasting effects, we collected purchase intention data from the same respondents several months after the labeling exposure in both studies. **Results**: Across both studies, increasing the proportion of products with a carbon label led liberals and centrists to choose lower-emission foods but had minimal or no impact on conservatives (although it never backfired). None of the other individual characteristics moderated the effects of labeling after controlling for political ideology. However, a young age, a low subjective socioeconomic position, and an urban residence indirectly improved responsiveness to labeling by predicting a more liberal political ideology. The labeling effects observed for liberals persisted for four months but not longer. **Conclusions**: These findings demonstrate the critical moderating role of political ideology and provide actionable insights to improve the targeting and design of sustainability interventions.

## 1. Introduction

Food systems are responsible for 25 to 42% of all human-caused greenhouse gas (GHG) emissions and are a significant cause of energy use. Most of these emissions are attributed to the land-based sector, while the rest arise from energy use, industry, and food waste [1,2]. Hence, climate change and sustainable food choices are deeply interconnected. In addition to solving environmental concerns, implementing sustainable food policies can help address economic [3] and social inequity [4].

Environmental or eco-labels, including those that disclose carbon emissions, are a popular intervention to nudge consumers to make more sustainable choices [5], particularly in the food domain, by allowing them to easily grasp the environmental impact of their choices. The link between consumer behavior and climate change is complex, and consumers often face difficulties in determining which behavior genuinely benefits the climate [6]. Eco-labels are a relatively simple and affordable policy measure to enhance transparency and confidence in sustainability-related product features [7,8].

A substantial body of prior research has documented the beneficial effects of eco-labeling on consumer beliefs and attitudes, including the evaluation of the products, their perceived healthiness, and purchase intentions [9,10]. Carbon labeling, in particular, has been shown to influence purchasing behavior by increasing awareness of a product’s carbon footprint and elevating the significance consumers assign to sustainability considerations [11]. According to the Carbon Trust, nearly two-thirds of consumers say they feel more favorable toward companies that actively reduce the carbon impact of their products [12].

However, evidence of the effectiveness of eco-labels on consumers’ actual behaviors is mixed [13]. Some studies have shown that eco-labels increased the sales of labeled products and led to a greater preference for climate-friendly meal options [11,14,15,16]. In particular, some studies found that carbon labeling led to an up to 3% reduction in carbon emissions [9,17,18]. Other studies, however, found no effect [19] or mixed results [20]. For example, studies conducted in worksite cafeterias found no measurable impact of carbon labels on purchases [21], GHG emissions, biodiversity loss, eutrophication, and water scarcity [19]. A systematic review by Potter et al. [22] revealed that over 20% of interventions testing different eco-labeling strategies failed to produce statistically significant changes in consumer behavior.

These inconsistent results suggest the presence of heterogeneity in consumer responses to eco-labeling. Research on nudges in general, not just eco-labels, has already suggested that differences in population characteristics, particularly socioeconomic ones, explain some of the conflicting findings about a nudge’s effectiveness [23]. Research has also shown that sustainable product choices vary by demographics, values, and lifestyles [24,25]. Consequently, we expect that, while some consumers are certainly responding to eco-labels as intended by shifting their purchases to products with a better environmental impact, other consumers are uninfluenced by eco-labels. Some consumers may even respond negatively to eco-labeling by choosing products with a worse environmental impact. For example, Waal et al. [26] suggested that sustainability claims could discourage purchases among consumers with lower environmental engagement.

The goal of this research is to examine individual differences in response to carbon footprint labels in food choices, expecting them to offer insights into the variability observed across existing studies. We examine the effects of seven theory-driven individual differences simultaneously: demographic characteristics (age, gender, and ethnicity), place of residence (categorized by their degree of urbanization), socioeconomic status (operationalized through educational attainment and subjective socioeconomic position (SEP)), and political ideology (from liberal to conservative). We then examine the psychological mechanisms underpinning these individual differences by exploring the role of political ideology and care for the environment. Finally, we examine whether the effects of labeling persist over the medium (four months) and longer-term (nine months).

In doing so, we aim to contribute to the existing body of research on how eco-labeling influences food choices while contributing to the debate on whether marketing interventions can reduce disparities [23,27,28]. Existing research on individual differences in response to labeling has focused on nutrition, not eco-labels [29,30]. In addition, existing research has focused on psychological outcomes, providing robust evidence that eco-labeling enhances consumer intentions to purchase sustainable products [31]. Given the high risk of socially desirable responses and self-presentation biases, it is important to study meaningful choices using incentive-compatible designs. Only a small subset of studies has investigated actual sales or choice data that would reflect real-world decision-making [14,32], and none has investigated the role of individual heterogeneity in response to eco-labels. In addition, many of the studies incorporated a high risk of bias, because participants could infer the purpose of the experiment. Also, some of them lack random sequence generation [22].

Another important limitation of prior studies is that they mostly considered only one or, at most, a few individual characteristics simultaneously. This poses a methodological challenge, as many of these factors are highly interrelated, making it difficult to isolate the effects of individual variables. Consequently, incorporating all relevant factors when analyzing behavioral outcomes will provide insight into whether specific effects remain significant while accounting for all of the other individual and demographic variables. The only exception in the sustainable behavior literature is the study by Frehner et al. [33], which accounted for multiple socio-demographic variables simultaneously but did not examine the effects of carbon labeling.

Finally, existing research on eco-labeling has focused on comparing two conditions: one in which no products are labeled and one in which all of them are, as in Reference [34]. In reality, eco-labeling is voluntary and is unlikely to become mandatory, especially in the food domain. As already seen for nutrition labels, the products with the highest ratings have a stronger incentive to display labels, while lower-rated products tend to avoid them. At first, consumers are likely to only encounter products with a good label. It is only over time that products with medium or poor labels will gradually adopt the labels following pressure from consumers or public opinion. This pattern, previously observed with nutrition labels, is unlikely to differ for eco-labels [35]. Consequently, we examine responsiveness to different levels of labeling, from cases when no product is labeled to cases where only the best products are labeled, and finally, to cases when all the products are labeled.

Overall, we extend previous research in a novel way by accounting for all individual characteristics simultaneously while examining the impact of a gradual increase in the proportion of carbon-labeled products on consumer choices.

### 1.1. Heterogeneity in Response to Food Labeling

#### 1.1.1. Demographics (Age, Gender, and Race and Ethnicity)

Age is the most frequently studied demographic variable in the context of eco-labeling. Research generally suggests that younger consumers exhibit a higher willingness to purchase carbon-labeled products [36]. For example, Shuai et al. [37] discovered that consumers aged 20 to 50 years are more likely to pay for low-carbon products, attributing this to younger consumers’ higher price sensitivity, while older adults express lower awareness of low-carbon products. Although some studies report no impact of age [38] or mixed results [39], most studies of real or incentive-compatible choices tend to find that younger consumers are more likely to purchase labeled products (e.g., [10,14,40]). The few studies that found the opposite result and concluded that age increases interest in eco-labels focused on beliefs or intentions, not behaviors [41,42,43]. Overall, we hypothesize that younger respondents will exhibit greater responsiveness to carbon-labeled products.

Research on gender-based differences provides tentative evidence that women have a higher likelihood of considering eco-labels [14,42,43,44,45,46] and choose these products more than men [40,45]. This difference occurs both in behavioral and psychological outcomes. A few field studies found no significant differences between female and male participants [9,14] and two recent studies found that men were more open than women to sustainable products when they are labeled [37,41]. Overall, however, we anticipate women will respond more favorably to carbon labeling.

Research examining eco-labeling responses across different ethnic groups remains limited. We identified only two studies that explicitly looked at how consumers from different racial or ethnic backgrounds react to labeling interventions. While numerous studies on eco-labeling have been conducted across various nations (e.g., China, Sweden, France, Poland, or the UK [31]) with substantial differences across them regarding label preferences [47], many have also focused on the United States (e.g., [10,42])—a country undergoing significant ethno-racial diversification [48]. Among the limited research available, Cholette et al. [42] found that across all groups, white students were the most likely to switch a price to a sustainability focus following exposure to an eco-label. However, another study found that African American participants expressed stronger endorsement of eco-labeling criteria in the food category than white respondents [49]. Based on the more recent findings [42] and given the simplified racial classification available in our data (i.e., white respondents vs. other races/ethnicities), we hypothesize there will be no significant effect of race and ethnicity.

#### 1.1.2. Place of Residency

Research on sustainable consumption in general, and eco-labeling in particular, has not explicitly examined the effects of living in an urban or rural setting. We could expect that urban consumers might react more favorably to eco-labeling thanks to the higher availability of sustainability-focused retailers [37,50]. Fan et al. [51] report that urban consumers generally have more knowledge, higher usage, and perceive that there are higher benefits of nutrition labels than rural residents. However, these studies were conducted in China, limiting their direct applicability to the context of the United States. Building on these findings, we predict urban consumers will exhibit a higher likelihood of choosing low-emission products compared to rural consumers when carbon labeling increases.

#### 1.1.3. Socioeconomic Status (Subjective SEP, Education, Income, Occupation)

Early studies on the role of socioeconomic status (SES) found that consumers with a low socioeconomic status understand nutritional labels less effectively than those with a high socioeconomic status and have difficulty using them to gauge the nutritional quality of food products [52]. However, more recent studies indicate that healthy eating nudges influence food choices similarly across socioeconomic groups [29,30]. Beyond nutritional labeling, studies found that respondents of high socioeconomic status [38,53] or middle-tier SES groups [39] are more likely to engage in pro-environmental behaviors. Part of this inconsistency may stem from different operationalizations of socioeconomic position; some studies rely on educational attainment, while others use subjective socioeconomic position like the MacArthur Scale [54]. However, prior findings regarding the effects of subjective socioeconomic position were obtained in the context of nutrition labeling, not eco-labeling. In addition, no study has specifically examined whether SES moderates the impact of eco-labeling.

Research on the role of education has found that higher educational attainment is associated with stronger responses to carbon labeling (e.g., [37]), with some exceptions finding no effect (e.g., [55]). Given the usually high correlation between education and income, this result confirms that socioeconomic status increases responsiveness to eco-labeling [31]. Socioeconomic status is sometimes inferred from occupation. For example, Zhao and Geng [36] found that students are the most willing to pay for carbon-labeled products. However, this result may be explained by age, not occupation. More generally, the relation between occupation and socioeconomic status is not always clear (e.g., it would be difficult to determine whether farmers are above or below retirees in terms of socioeconomic status). Therefore, we cannot make a formal hypothesis regarding the role of occupation.

#### 1.1.4. Political Ideology

Recently, there has been a growing interest in the relationship between sustainable consumption and consumers’ political ideology [56]. On average, liberals tend to embrace environmental messages, while conservatives show skepticism toward regulatory approaches [57]. Only one study examined the moderating role of political ideology in a real-choice experiment in response to eco-labeling [45]. It found that conservatives are less likely to purchase environmentally labeled products in comparison to liberal consumers. However, this study focused on energy-efficient light bulbs. Although no research has been conducted on the food sector, these results suggest that liberals would be more responsive to eco-labels than conservatives.

#### 1.1.5. Hypotheses and Overview of Studies

Based on previous findings, we can state the following eight hypotheses: As the proportion of carbon-labeled products increases, consumers who are younger, female, white, and urban are more likely to choose low-emission food products compared to consumers who are older (H1), male (H2), non-white (H3), and rural (H4). The same pattern holds for individuals who are higher-income, are in a higher subjective socio-economic position (SEP), have a high level of education, and are liberal in comparison to those who are lower-income (H5), have a lower SEP (H6), have a lower level of education (H7), and are conservative (H8).

In addition, we hypothesize that political ideology and care for the environment mediate, as a serial mediation, the effects of the other individual characteristics on responsiveness to carbon labeling.

We test these hypotheses in two pre-registered incentive-compatible choice studies. All the data, code, pre-registrations, and survey stimuli are available on Researchbox #3989. The first study examines the effects of carbon labeling on the choice of instant meal products in an online grocery store. By asking participants to make two choices among 10 cut fruit options, first without carbon labels and then with varying levels of carbon labels, the second study examines the effects of carbon labeling on choice changes. Study 2 also forces participants to make a tradeoff between their preferences and carbon emissions.

In both studies, we examine all individual characteristics factors simultaneously. Finally, we aim to understand the persistence of the effects by examining their effects on consumers’ intentions to purchase products with low or high carbon emissions four and nine months after the main experiment.

#### 1.1.6. Statistical Analyses

All statistical analyses were performed using R software (version 4.4.3). We employed a series of linear regression models to investigate the associations between the individual factors and the dependent variables, including ‘Choice of lower-emission products’ (Study 1), ‘Shift to lower-emission products between the first and second choice’ (Study 2), and ‘Intentions to purchase A or B and D or E products (Study 3)’. To assess indirect effects, we conducted a serial mediation analysis using the lavaan package in R [58]. This approach allowed us to examine whether the impact of socio-demographic factors on the shift to lower-emission products was transmitted through political ideology and care for the environment.

All predictor variables were either mean-centered or effect-coded, depending on their scale, to ensure the interpretability of the model coefficients and potential multicollinearity problems. All statistical tests were two-tailed, with results considered statistically significant at an alpha level of 0.05. Additional information on the descriptive analyses and the operationalization of measures is provided in each individual Method chapter of the following studies.

## 2. Study 1: Heterogeneity in Response to Eco-Labeling

### 2.1. Method

#### 2.1.1. Participants

As previously mentioned, we only recruited American consumers who purchase groceries online, have bought instant meals from grocery stores at least once, and do not follow a vegetarian diet. The participants were recruited in two stages. First, we recruited respondents to make an incentive-compatible online grocery store purchase under varying levels of carbon labeling, as described below. After eliminating participants who failed an attention check (which asked them to recall the product category they had shopped from), we obtained 1303 responses. In the second stage, we contacted these participants weeks after the first study to measure the individual characteristics data and their intentions to purchase products with a good or bad carbon label.

Conducting the study in two stages ensured that the answers to one stage were not influenced by the answers to the other. For example, measuring political ideology weeks after the food choice rather than before ensured that respondents would not change their choices to be consistent with their political ideology. It also allowed us to measure the longer-term effects of exposure to carbon labeling.

We merged the responses collected in the two stages, obtaining a final sample size of 715 participants (58% female; M_age_ = 44 years; SD_age_ = 13 years). We did not find systematic differences between the respondents who participated in the two studies and those who only participated in the first study regarding their product choices and responsiveness to labeling (see details in Section S1 in the Supplementary Materials).

#### 2.1.2. Design

Study 1 employed a between-subject, incentive-compatible design with six labeling conditions. In the first condition, none of the products was accompanied by a carbon label. In the second condition, only products with an A rating (those with the lowest carbon emissions) were labeled. In the third condition, products with an A or B rating were labeled. In the fourth condition, products with an A, B, or C rating were labeled. In the fifth condition, products with an A, B, C, or D rating were labeled. In the sixth condition, products with an A, B, C, D, or E rating were labeled, meaning that all products were labeled. The labeling intervention was designed to capture the gradual introduction of eco-labeling that is likely to occur as more products are labeled and more consumers expect products to be labeled. This intervention allows us to obtain a more representative depiction of labeling, but the proportion of labeled products is not germane to our hypotheses, which focus on individual differences in responses to labeling in general. For this reason, we examine individual differences in how people respond to increasing the proportion of products labeled. We did not compare specific conditions (e.g., when only products with an A or B rating are labeled compared to when all products are labeled).

To label the products, we used the My Emissions front-of-pack carbon footprint rating system, which follows a color-coded traffic light system to rate a product’s environmental impact. It ranks products from A (very low impact) to E (very high impact) based on their carbon footprint per 100 g. The label is used by companies such as Just Eat Takeaway.com and was displayed on the menu boards at the 2024 UEFA Champions League Final [59].

Individual carbon emissions and the corresponding label were calculated using the open-access My Emissions food carbon footprint calculator [60] based on the composition of each product. These estimates are based on a holistic life cycle approach, accounting for emissions from food production to disposal.

#### 2.1.3. Procedure

Respondents were recruited on Prolific and invited to participate in a study about food. Participants received instructions on how to choose one instant meal in an online grocery store (Howe’s Grocery) among twenty instant meal products sold in a large US grocery store. An advantage of this online grocery tool is that it includes products sold in American supermarkets that North American consumers are likely to be familiar with [61]. The products included instant pasta meals (e.g., a box containing dry pasta and a mac and cheese sauce packet) or microwaveable meals like chili or beef stroganoff. Respondents were told that some products might be accompanied by a color-coded five-category carbon footprint label with ratings ranging from A (best, in green) to E (worst, in red). To prevent customers from focusing their attention on carbon labeling, they were also told that some products might have a nutrition label (no product did). To create incentive compatibility, they were informed that 15 randomly selected participants would receive the product they had chosen. In the end, we gave the randomly selected participants a monetary bonus to allow them to purchase the product on their own.

**Figure 1:**
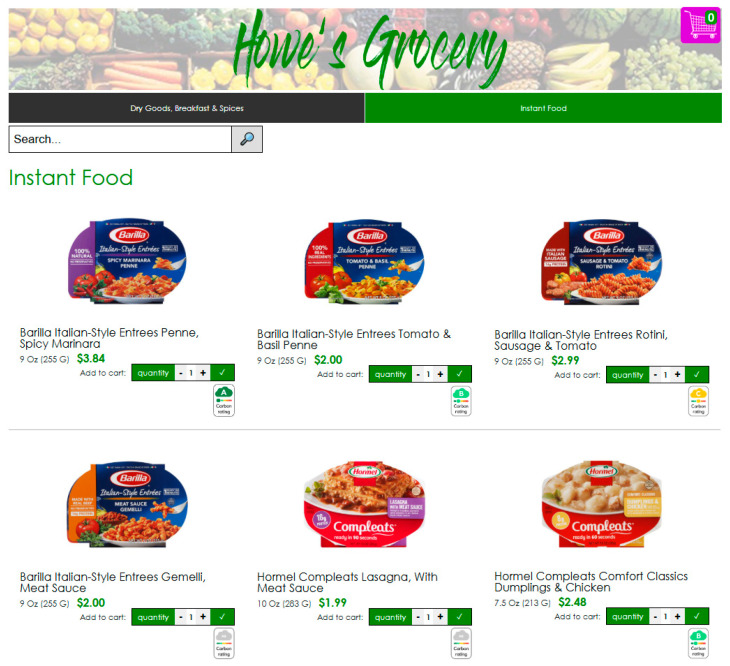
Study 1: Stimuli example.

Participants were then redirected to the online grocer. Figure 1 shows part of the first screen of the interface, including labeled and non-labeled products. Depending on the size of their screen, respondents had to scroll down three to four screens to see all 20 instant meal options. The site showed the packs, alongside the full name of the product (e.g., “Barilla Italian-Style Entrees Tomato & Basil Penne”), its price (using the actual price of a US retailer), and its weight, as well as an interactive shopping cart icon. Because eco-labels can also draw attention to products on the screen, in addition to providing information about their carbon footprint, all the products were accompanied by a logo of the same size stating “NR” for “not rated” (see Figure 1).

Participants could either add a product to the cart directly or first click on the package to reveal detailed composition and nutrition information. Upon finalizing their selection, participants proceeded through the checkout and were redirected to the survey, where they answered additional questions.

#### 2.1.4. Measures

Choice of lower-emission product (CLE). Consistent with previous studies looking at behavioral outcomes, we measured the sustainability of the respondents’ choice using a five-point scale ranging from 1 = choice of product with an E rating (highest carbon emissions) to 5 = choice of a product with an A rating (lowest carbon emissions).

Socio-demographic variables. To obtain comprehensive demographic information beyond age and gender, we asked respondents to indicate their level of education on a six-point Likert scale (1 = “Middle school or less”; 2 = “High school (without A-levels)”; 3 = “High school diploma (A-levels)”; 4 = “Completing the first or second year of college (freshman or sophomore)”; 5 = “Undergraduate degree (BA/BSc/other)”; 6 = “Graduate degree (MA/MSc/MPhil/other) or higher”), the level of urbanization of the place of residency on a five-point Likert scale (1 = “In the countryside (rural area)”; 2 = “Village”; 3 = “Medium-sized or small town”; 4 = “Suburb of a large city”; 5 = “Large city”), and their level of subjective socioeconomic position. For the latter, we utilized the 10-point MacArthur Scale of Subjective Social Status [54], in which the top of the ladder (10) represents people in a society who perceive themselves as best-off (e.g., in terms of education and income), whereas the bottom of the ladder (1) represents those who consider themselves worst-off.

We also collected income information using a seven-point Likert scale (ranging from 1 = “Less than $10,000” to 7 = “More than $60,000”). However, we excluded this predictor from all analyses after observing that more than 50% of respondents grouped themselves in the highest income bracket, which is not realistic. Likewise, the correlation between income and education was only 0.35. This suggests that some Prolific respondents inflated their income, possibly to be eligible for higher-paying surveys.

Political ideology. Respondents rated their political ideology on a seven-point Likert scale from 1 (very liberal) to 7 (very conservative). This single-item measure was widely used in prior research and shows a high predictive validity [62].

Occupation. Respondents reported their occupation by choosing among the following options: “Farmer/Artisan/Shopkeeper or Entrepreneur”, “Executive/Professional”, “Middle management”, “Employee”, “Student”, and “Prefer not to answer”.

### 2.2. Results

We estimated a linear regression with the choice of lower-emission products (measured from 1 to 5) as the dependent variable. The independent variables are the proportion of products labeled (from 0 to 100%), the seven individual factors described above, and their interaction with labeling while controlling for occupation. As Table 1 shows, the coefficient for labeling is positive and statistically different from zero (B = 0.13, *p* < 0.05). This means that, as the proportion of labeled products increases, consumers shift to lower-emission products. There was only one statistically significant main effect among the seven individual characteristics: people with a higher subjective socioeconomic position chose lower-emission products than those with a lower subjective position (B = 0.12, *p* < 0.05).

### 2.3. Discussion

Overall, Study 1 shows that political ideology is the only of the seven theory-driven factors used to moderate the effects of carbon labeling on sustainable product choices. This moderation primarily influences the choice of low-emission products over the D or E products. In addition, although labeling leads to a stronger shift to lower-emission products for liberals than for conservatives, this effect does not backfire for conservatives, whose choices are unaffected by carbon labeling.

This study provides valuable insights into the effects of carbon labeling across different groups of consumers; nevertheless, the design presents a potential limitation. Because we used the real GHG emissions and prices of the 20 instant meal products, the effects of labeling might be driven, in part, by the particular brands, the meal composition, or the price of the low- and high-emission products in the study.

To address this potential issue, we conducted another study in which product preferences were decoupled from emission levels and price. Accordingly, the preferred products were assigned higher CO_2_ emissions and prices. Furthermore, while participants in Study 1 only made one choice, those in Study 2 made two sequential choices, one among unlabeled products and a second among the same products with varying degrees of labeling. This procedure provides more control, as each participant serves as their own control. It also enables an assessment of the source of the new choices for low-emission products. For example, it allows us to determine whether the new choices of A or B products come from C products or from D or E products. Finally, we aim to determine if the effects can be replicated in a different product category.

The parameters of interest are the interaction effects between labeling and the individual-level factors. Only the interaction with political ideology was statistically significant (B = −0.06, *p* < 0.05). The negative coefficient indicates that labeling becomes less effective as political ideology shifts to the right.

**Figure 2:**
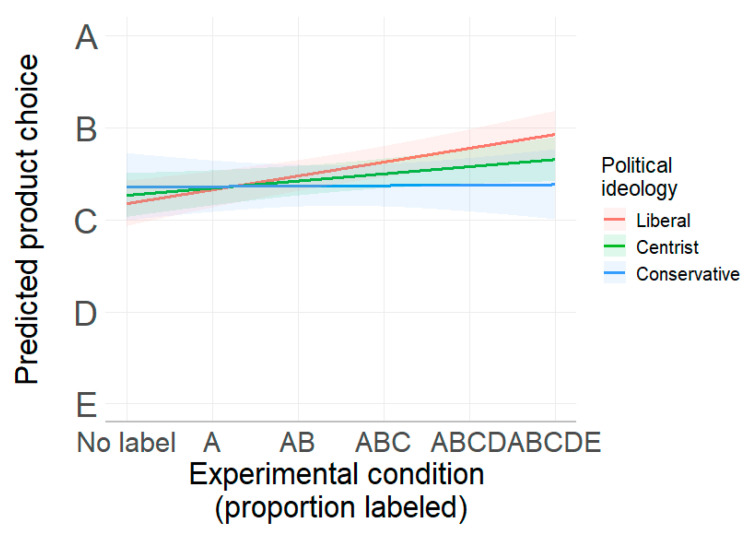
Predicted product choices (from lowest (A) to highest (E) emissions) for liberals, centrists, and conservatives in Study 1.

This can be seen in Figure 2, which plots the product choices predicted by the regression at three different levels of the 1 to 7 political ideology scale at 2 (liberals), 4 (centrists), and 6 (conservatives), while holding all other covariates constant. Figure 2 shows that carbon labeling works best among liberals. As the proportion of labeled products increases, liberals are the most likely group to shift to lower-emission products. A test shows that the slope is statistically significant for this group (B = 0.25, *p* < 0.001). The model predicts a weaker but still statistically significant improvement for centrists (B = 0.13, *p* < 0.05). In contrast, labeling has no effect on conservatives (B = 0.009, *p* = 0.93). Figure 2 also shows that the choices of the three groups are similar when no product is labeled but diverge as the proportion of labeled products increases.

**Figure 3:**
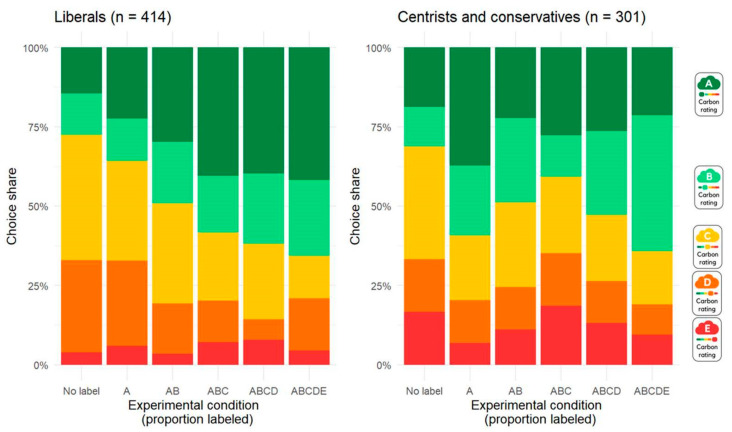
Distribution of chosen product types (from lowest (A) to highest (E) emissions) by labeling conditions and political ideology in Study 1.

To study the effects of labeling products in more detail, Figure 3 displays the entire distribution of choices (from A to E) for consumers categorized into two groups using a median split: liberals (from 1 to 3 on the political ideology scale) and centrists and conservatives (from 4 to 7 on the political ideology scale). The left panel of Figure 3 shows that, as the proportion of labeled products increased from none to all, liberals increasingly chose low-emission products (A or B products). When no products are labeled, liberals select 27% of A and B products, whereas this proportion rises to 66% when all products carry a carbon label. In contrast, the right panel reveals no distinct pattern among centrists and conservatives, suggesting that labeling had little effect on this group.

Figure 3 also shows that, at the baseline level, when no products are labeled, liberals, on the one hand, and centrists and conservatives, on the other hand, show a similar distribution of product choices, indicating that initial product preferences were comparable across political ideology groups. This suggests that the results are not driven by idiosyncratic preferences in each group for products with different carbon emissions.

## 3. Study 2: Heterogeneity in Changes in Responsiveness to Eco-Labeling

### 3.1. Method

#### 3.1.1. Participants

As pre-registered, we recruited American consumers who occasionally or regularly purchase fresh-cut fruits from the grocery store. Following the same procedure as in Study 1, we began with an initial choice study (n = 2058) and collected individual data about the participants later, yielding 1233 respondents (49% female; M_age_ = 44 years; SD_age_ = 14 years). As in Study 1, there were no systematic differences between the participants who completed both waves and those who only participated in the first wave (see details in the Supplementary Material Section S2).

#### 3.1.2. Design

We implemented an incentive-compatible design with four labeling conditions. In the first condition, none of the products was accompanied by a carbon label. In the second condition, only products with an A or B rating were labeled. In the third condition, products with an A, B, or C rating were labeled. In the fourth condition, products with an A, B, C, D, or E rating were labeled, meaning that all products were labeled.

To eliminate product-specific confounds, we first asked participants to rank 12 fresh-cut fruits by order of preference. After eliminating their two least-preferred products (those ranked 11 and 12), the worst (E) rating was assigned to each respondent’s top two ranked products, while the D rating was assigned to the products ranked 3 and 4, the C rating to those ranked 5 and 6, the B rating to the products ranked 7 and 8, and the A rating to the products ranked 9 and 10. Also, the highly ranked (preferred) products were displayed at a lower price. This ensured that each respondent was forced to make a tradeoff between their own preference and carbon emissions.

Participants then completed two sequential choice tasks. In the first task, they chose one fresh-cut fruit option from their ten favorite ones. They were given photos of the products, prices, and weight information, but no carbon labels were displayed. They then made a second choice among the same assortment but with, depending on the condition, from zero to all products labeled, allowing us to measure changes in choice following carbon label exposure. See Supplementary Material Figure S1 for an example of the stimuli for each condition. The study also included another manipulation involving how the environmental footprint of the cut fruits was interpreted. This intervention is irrelevant to our purposes but is controlled for in the analyses.

Finally, all participants completed an attention check and answered additional questions about their attitude toward the environment.

#### 3.1.3. Measures

Like in Study 1, we measured carbon emissions on a five-point scale from 1 = choice of product with an E rating (highest carbon emissions) to 5 = choice of a product with an A rating (lowest carbon emissions). To measure the change in carbon emissions between the first (unlabeled) and second (labeled) choice, we subtracted the score in choice 2 from the score in choice 1. This difference in score variable was measured on a scale of −4 to +4. A difference score of 0 signifies that respondents chose the same product type in the first and second choices. A positive score indicates that respondents shifted to a lower-emission product in the second (labeled) choice. The maximum score, +4, indicates that the respondent shifted from an E (1) to an A (5). A negative choice is a shift to higher-emission products (the minimum, −4, indicates that the respondent shifted from an A = 1 to an E = 5).

All the socio-demographic variables, as well as the measures for political ideology, were the same as in Study 1. As in Study 1, the correlation between income and education was only 0.32, leading us to exclude this from the analyses. In addition, at the end of the survey, participants completed the Brief Ecological Paradigm scale [63], which measures care for the environment using items such as “humans are severely abusing the environment” on a five-point Likert scale, ranging from 1 (strongly disagree) to 5 (strongly agree).

### 3.2. Results

We used the same regression as in Study 1 but with the shift to lower-emission products between the first and the second choice as the dependent variable. The independent variables captured gradual labeling conditions, with only an additional control variable for the information condition. To provide a direct comparison to the results of Study 1, we tested the effects on the (second) choice of lower-emission product (label E = 1 to A = 5). The results of this analysis yielded the same conclusions. They are available in Table S1 in the Supplementary Material.

The results shown in Table 2 reveal a strong and significant main effect of labeling and political ideology on the shift to lower-emission products. As in Study 1, people shifted to lower-emission products as labeling increased. Additionally, conservatives exhibited a lower likelihood of shifting to lower-emission products when they were labeled. Note that, given the repeated-measures design of Study 2, the dependent variable already measured shifts in choices to lower-emission products between the first scenario (when no products are labeled) and the second scenario (when a varying proportion of products are labeled). Thus, the main effect of political ideology can be interpreted as a lower responsiveness to labeling for conservatives than for liberals.

Moreover, we identified two significant interaction effects involving labeling. First, unlike in Study 1, there is another significant interaction with gender: women respond more strongly to labeling than men. Second, as in Study 1, conservatism reduces the positive effects of carbon labeling on the shift to lower-emission products. This can be interpreted as follows: For liberals, increasing the proportion of labeled products further increases the shift to lower-emission products in the second choice. This further increase in the choice of lower-emission products is less pronounced for conservatives.

**Figure 4:**
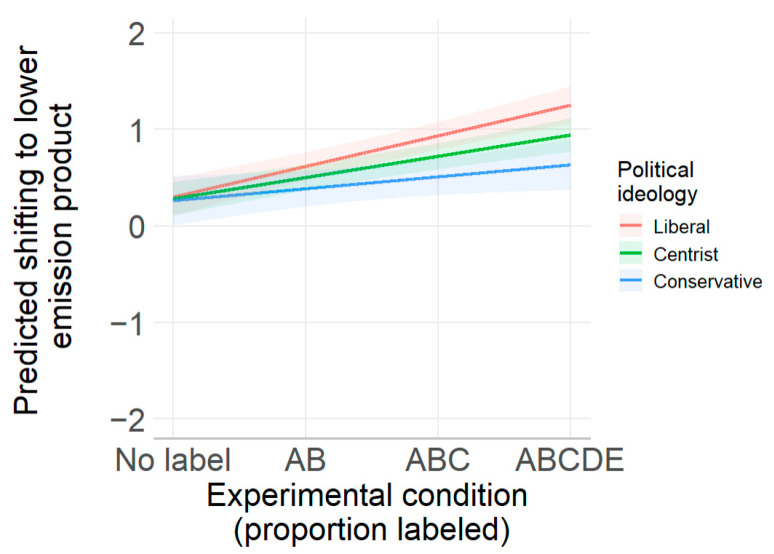
Predicted shift to lower-emission products (from lowest (A) to highest (E) emissions) for liberals, centrists, and conservatives in Study 2.

The moderating effect of political ideology can be seen in the spotlight analyses shown in Figure 4, which plots the shift to lower-emission products predicted by the regression at three different levels of the 1-to-7 political ideology scale at 2 (liberals), 4 (centrists), and 6 (conservatives), while holding all other covariates constant. Figure 4 shows that liberals are more likely to switch to lower-emission products as carbon labeling increases (B = 0.36, *p* < 0.001). The same shift occurs for centrists, but to a lesser degree (B = 0.25, *p* < 0.001), and for conservatives to an even lesser degree (B = 0.14, *p* = 0.06). As in Study 1, there was no backfiring effect among conservatives. As in Study 1 as well, the three groups obtained similar results in the no-label condition: all groups exhibited minimal shifting across the two decisions.

**Figure 5:**
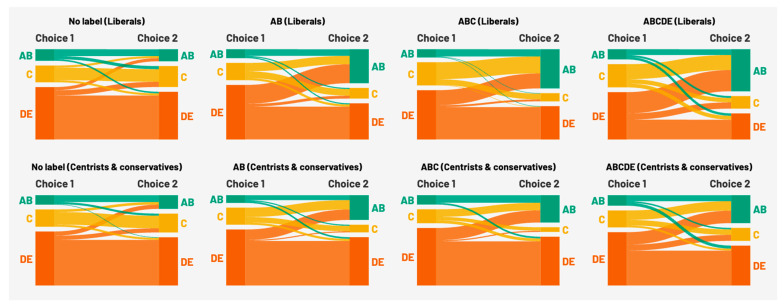
Changes in the distribution of chosen product types (from lowest (A) to highest (E) emissions) by labeling conditions and political ideology in Study 2.

As in Study 1, we explored the effects of labeling on the distribution of chosen products in more detail. The alluvial plots in Figure 5 show, on the left, the distribution of chosen products in the first and second choices, grouped into three categories: low-emission (A and B) products, middle-emission (C) products, and high-emission (D and E) products. They also show where the gains and losses in these choices come from.

As in Study 1, the distribution in the no-labeling condition is comparable across the two groups, liberals (top row of Figure 5) and centrists and conservatives (bottom row of Figure 5). Both groups chose mostly D and E products, which were their preferred products in each construction, and there was very little change between the first and second choices. However, increasing the proportion of labeled products (progressing from the left chart to the rightmost chart) led liberals (top row) to shift to AB products and away from DE products more than centrists or conservatives (bottom row).

Unlike in Study 1, where political ideology influenced the share of low-emission (AB) products more than that of high-emission (DE) products, political ideology influences the choice of both low- and high-emission products in Study 2. The alluvial plots show that the gains in AB products not only come from middle-emission (C) products but also from high-emission (DE) products.

#### Serial Mediation Model: Role of Political Ideology as a Mediator

Across both studies, political ideology consistently emerges as the strongest predictor of label responsiveness. Still, it is possible that other factors influence responsiveness to labeling indirectly, through their impact on political ideology. Additionally, we examine whether the effects of political ideology are not themselves mediated by care for the environment.

To test these paths, we implemented a serial mediation model, where socio-demographic factors serve as independent variables, political ideology functions as the first mediator, and environmental concern acts as the second mediator. The dependent variable remains label responsiveness, measured in the same way as in the regression analyses. The control variables are occupation, a binary variable accounting for information manipulation, and the labeling proportion.

**Figure 6:**
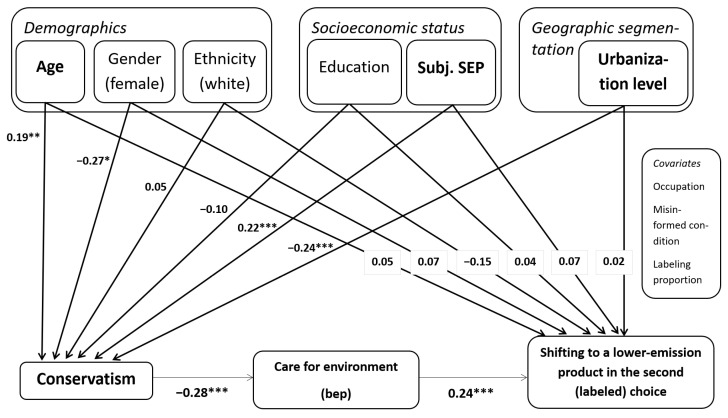
Serial mediation model, Study 2, * *p* < 0.05; ** *p* < 0.01; *** *p* < 0.001.

The mediation analyses reveal that age, subjective SEP, and the level of urbanization significantly influence label responsiveness but indirectly, through political ideology, which, in turn, influences care for the environment and the likelihood of shifting to a lower-emission product, as shown in Figure 6. Specifically, age is associated with increased conservative tendencies, which in turn reduces the shift to low-emission products when products are labeled (indirect effect: β = −0.013; *p* = 0.011). Similarly, a higher perceived subjective economic position is linked to greater conservatism, which significantly reduces the shift to low-emission products when products are labeled (indirect effect: β = −0.015; *p* = 0.007). Conversely, Americans residing in urban rather than rural areas tend to be less conservative and thus more likely to shift to lower-emission products when they are labeled (β = 0.016; *p* = 0.004).

### 3.3. Discussion

Taken together, Study 2 replicates the results from Study 1, while extending the analysis to a new product category in a design in which product preferences are independent of brand and must be traded against CO_2_ emissions and by measuring the actual shift in respondents’ product choices when products are labeled. As in Study 1, increasing the labeling proportion leads liberals to choose more sustainable products, but this effect is weaker for centrists and disappears for conservatives. As in Study 1, labeling does not backfire among conservatives.

In addition, we found an interaction between labeling and gender, where women were more likely than men to switch to low-emission products as labeling increased. This result aligns with the prior research on gender differences in sustainable consumption [42,43,45,46]. However, we did not observe a main effect of gender, even when removing all interaction terms (B = 0.08, *p* = 0.37). Also, there was no interaction effect involving gender in Study 1. Given the lack of replication, more research is necessary to determine whether gender reliably influences responsiveness to carbon footprint labels.

In Study 2, we further examined the structure of the relationship between individual characteristics by examining the mediating role of political ideology and care for the environment. Consistent with prior research, older people [64], those with a higher subjective socioeconomic position (SEP) [65], and those with a rural place of residency [66] were more likely to be conservative. In turn, conservatism significantly reduces individuals’ care for the environment, confirming earlier results (e.g., [67]). The serial mediation model findings demonstrate that, despite there being no significant direct effects of other individual characteristics, age, subjective SEP, and the level of urbanization indirectly shape the responsiveness to labeling through political ideology.

In the absence of information about political orientation, companies can use age, place of residence, and subjective socioeconomic position as a proxy for political orientation and target people who are younger, live in cities, and have a lower subjective socioeconomic position. If information about political ideology is available, however, these other variables are unnecessary.

Importantly, the two indirect effects of age and place of residence on label responsiveness are in the same direction as the hypothesized direct effects, with higher responsiveness to labeling for younger people and for people living in urban settings. For subjective SEP, however, the indirect effect is in the opposite direction than the hypothesized direct effect, which argued, based on prior research on socioeconomic status, that people with a higher subjective socioeconomic position would be more responsive to labeling. What we found instead is that people with a high subjective socioeconomic position, because they tend to be more conservative, are—overall—less responsive to labeling.

While these findings are promising, an important question remains: Do these positive shifts toward low-emission product choices, particularly among liberal consumers, persist in the medium- or long-term? We address these questions in Study 3.

## 4. Study 3: Medium- and Long-Term Effects of Exposure to Carbon Labeling

### 4.1. Method

To capture whether the effects of exposure to carbon labeling persist, we used purchase intention data collected in the second stage of each study, nine months after exposure to carbon labeling in Study 1 and four months after exposure to carbon labeling in Study 2 (medium-term effects).

In both cases, we asked respondents to rate their intentions to purchase products from the same category as in the main study (instant meals in Study 1 and cut fruits in Study 2) with A or B carbon labels or with D or E carbon labels on a five-point Likert scale, ranging from 1 (definitely will not) to 5 (definitely will). We also asked respondents about their intentions to purchase a product with a C carbon label. However, given the ambiguity when classifying these products as low- or high-emission, we focused on purchase intentions for A or B products (low-emission) and for D or E products (high-emission).

### 4.2. Results and Discussion

We regressed intentions to purchase each type of product on the same independent variables as in the other analyses. Table 3 shows the results of the four regressions. The first two regressions show the effects of exposure to labeling on intentions to purchase low-emission products (those with an A or B label), whereas the last two show the effects on intentions to purchase high-emission products (those with a D or E label). In each case, we report the medium-term effects (for cut fruits in Study 2) and long-term effects (for instant meals in Study 1).

Table 3 shows no significant main effect of labeling exposure on either medium- or long-term intentions. As expected, conservatives have lower intentions to purchase low-emission products and higher intentions to purchase high-emission products for both cut fruits and instant meals. Importantly, there is a statistically significant interaction effect between political ideology and labeling proportion on intentions to purchase low-emission products, but only for cut fruits (in the medium term). This indicates that the medium-term effects of labeling are lower for conservatives than liberals.

To interpret this interaction effect, we performed a floodlight analysis to help identify the regions of significance. It revealed that carbon labeling had a statistically significant positive effect on the medium-term intention to purchase A- or B-labeled products when political ideology is below 2.2 on the seven-point scale of political ideology (where 1 = very liberal and 7 = very conservative). For liberals, intentions to purchase low-emissions cut fruits persisted in the medium term after initial exposure to carbon labels and increased with the proportion of products labeled.

In summary, carbon footprint labeling significantly enhances intentions to purchase A- or B-labeled products among liberals four months after the initial exposure to the carbon labels. However, no long-term effects are observed when purchase intentions are measured nine months later. These differences are tentative since they may stem from variations between the two studies, such as the choice of product, and not just in the time elapsed between the exposure to the labels and the measure of purchase intentions.

## 5. Performance Analysis: Cohen’s *d*

To examine the magnitude of change across the proportion of labeling for each dependent variable, we computed Cohen’s *d* for all studies [68]. We compared the treatment condition (fully labeled) and the control condition (no label). The choice of lower-emission product showed a medium-sized effect size for Study 1, with Cohen’s *d* = 0.57, SE = 0.13, and 95% CI [0.31,0.84], and Study 2, with Cohen’s *d* = 0.60, SE = 0.08, and 95% CI [0.45,0.76]. In Study 2, the shift to lower-emission products between the first and second choice was also associated with a medium effect size (Cohen’s *d* = 0.61, SE = 0.08, and 95% CI [0.45,0.77]).

In contrast, we observed small to very small effect sizes for purchase intentions. For intentions to purchase A or B products, we detected very small effects in the medium-term (Cohen’s *d* = 0.13, SE = 0.08, and 95% CI [−0.03,0.29]) and the long-term period (Cohen’s *d* = −0.07, SE = 0.13, and 95% CI [−0.33,0.18]). The same pattern was found for intentions to purchase D or E products, with even smaller effects in the medium-term (Cohen’s *d* = 0.03, SE = 0.08, and 95% CI [−0.13,0.18]) and the long-term period (Cohen’s *d* = −0.03, SE = 0.13, and 95% CI [−0.28,0.23]).

These findings highlight that the effect of the increase in the proportion of labeling was more pronounced for choice settings, compared to the small effects observed for intentions.

## 6. General Discussion

In this article, we explore how individual characteristics—particularly political ideology, age, gender, ethnicity, occupation, urbanization level, and two measures of socioeconomic status (education and subjective socioeconomic position)—shape responsiveness to carbon footprint labeling. Extending previous work, we examined all individual factors simultaneously while studying the effects of a gradual increase in the proportion of products that are labeled.

Two incentive-compatible studies, one conducted in an online grocery store with the full assortment of instant meal products sold in a US supermarket chain and the other using a design forcing consumers to make a trade-off between their preferences for cut fruits and those with a lower carbon footprint, demonstrate that eco-labeling is effective. Consistent with prior studies, carbon labeling leads participants to choose products with lower carbon emissions. In addition, the effectiveness of carbon labeling increases as the proportion of labeled products increases from just labeling the lowest-emission products to labeling all products.

Both studies also show that the effectiveness of carbon labeling decreases with conservatism. Carbon labeling is most effective among liberals, leading them to select lower-emission products and to switch from preferred but high-emission products to less-preferred products with lower emissions. The effects are weaker but still statistically significant among centrists. No such effects were observed among conservative consumers. Still, carbon labeling does not backfire among conservative consumers (it does not lead them to choose higher-emission products). Furthermore, once political ideology is included, none of the other socio-demographic variables influence responsiveness to carbon labeling. The only exception is the effect of gender, which was significant in one study but not in another.

Although socio-demographics, socioeconomic status, and place of residence do not directly moderate the effects of labeling once political ideology is accounted for, some of these variables have an indirect effect through their effects on political ideology. Specifically, we find indirect effects of age, the level of urbanization, and subjective socioeconomic position via political ideology (Study 2). While no long-term effects of exposure to carbon labeling emerge, we find that they persist over the medium term (four months), but only among liberal Americans (Study 3).

### 6.1. Research Implications

Given the urgent need for global environmental actions, additional research is needed to more fully understand when and why eco-labeling works. First, our results suggest that the mixed results found in the literature may be partly explained by sample composition. For example, the positive results found in most studies may be caused by their reliance on university students or young participants, who tend to be more liberal and thus more responsive to eco-labeling (e.g., [14]). It would be useful to conduct a meta-analysis of prior work that would control for differences in sample composition. Such a meta-analysis could also explore the interaction effects among these individual factors. As intersectional approaches gain increasing relevance in marketing, overlapping rather than isolated social categories should be acknowledged and investigated in future studies [69]. For example, future research should examine the interplay of different individual characteristics, such as the joint effects of political ideology and gender. Based on this, future experimental studies regarding different eco-labels, particularly those utilizing real-choice settings, should strive to incorporate as many individual characteristics as possible.

Our results show that the effects of political ideology are linked to care for the environment. Given its importance in our results and its complex nature, additional research should focus on understanding why political ideology influences care for the environment in the first place. One possible explanation may be that, as suggested by research on Fair Market Ideology [70], conservatives tend to moralize free market outcomes, assuming that they are inherently fair, and hence that labels, like other forms of governmental interventions, may not be necessary. Another explanation is provided by the Moral Foundations Theory [57], which argues that conservatives focus on values like authority and loyalty, whereas liberals prioritize care and fairness. Accordingly, conservative consumers may be more skeptical of regulatory interventions and prefer market-driven solutions, while liberals are more likely to support ecolabels as necessary guides for ethical consumption. A third and orthogonal explanation may be that liberals, but not conservatives, perceive carbon labels as reliable indicators of a product’s environmental impact.

Subsequent research may also take a broader definition of sustainability and look beyond GHG emissions. Although carbon footprint considerations are essential, they are not the sole criterion when assessing the effectiveness of eco-labeling. Similarly, future research could examine whether carbon labeling changes the supply of products rather than the demand for existing products. Carbon labels may promote product reformulation designed to lower carbon emissions. Retailers could also choose to delist products with a bad label.

Although we consider this study as a solid first step in incorporating consumer characteristics into eco-labeling research, it is not without limitations. Future research should extend these effects by examining the role of political ideology and other individual characteristics in countries other than the United States. Additionally, research should seek to design interventions beyond labeling to better reach the large segment of moderate and conservative shoppers, particularly in expanding product categories. Another important avenue for future research would be to measure consumer preferences for carbon or other types of labeling in general. For example, it would be interesting to know whether consumers would prefer to shop at retailers that display carbon footprint labels. Our findings imply that liberals may be willing to pay for carbon labels.

### 6.2. Managerial Implications

These results have practical importance. First, let us consider the “most favorable” condition: liberal consumers (with a score of 1 on the 1–7 point scale of political ideology), who are female, living in a large city, older (88 years old), non-white, have the highest SEP (10 on the 1–10 point scale of SEP) and are well-educated (holding a graduate degree or higher). Among this group, exposure to 100% carbon labeling would be predicted to lead to a two-carbon label level improvement (e.g., from a C to an A label) for 50% of the respondents and to a one-level improvement for the remaining 50%. In contrast, among those in the “least favorable” scenario, only 37% moved up by one label level, while 63% did not change their selection. This group, composed of 19-year-old, white, rural (in the countryside), low-SEP (1 on the 1–10 point scale of SEP), and less-educated (middle school or less) individuals with very conservative attitudes (on a seven on the 1–7 point scale of political ideology), exhibits minimal responsiveness to carbon labeling, with a label switch of only 9.2% being the maximum possible change.

From a practical perspective, our findings suggest that simply labeling a product’s environmental impact is unlikely to sway conservative or centrist consumers. Brands with a centrist or conservative customer base are less likely to experience sales shifts as a result of carbon labeling compared to those serving more liberal customers. Similarly, retailers should prioritize labeling in regions with a predominantly liberal customer base.

Our results also suggest that marketing strategies to encourage sustainable choices should be adapted to align with consumer ideologies. Food chains with a more liberal-leaning customer base are more likely to encourage consumers to select their products by promoting carbon labeling. Implementing this approach may positively impact sales, and the effects might endure beyond the decision at the point of sale, particularly in the short-term period, a few months after the purchase of the carbon-labeled product.

Conversely, brands with a more conservative-leaning audience may not benefit from eco-labeling and might instead explore different ways to nudge their customers. When targeting conservatives, emphasizing CO_2_ emissions may be less beneficial than focusing on more value-aligned messages, such as national preferences, which would also promote the purchase of local and potentially lower-emission products. Conversely, the absence of backlash against high-emission brands with a conservative-leaning customer base suggests that these brands could enhance their reputation through transparency, by labeling their products, without experiencing adverse commercial effects.

## 7. Conclusions

This research investigates heterogeneity in consumer responses to carbon footprint labeling in food choices. By simultaneously analyzing multiple individual characteristics across two incentive-compatible studies, we provide robust evidence that the effectiveness of carbon labeling is strongly moderated by political ideology, with liberals exhibiting the most pronounced behavioral shifts and conservatives showing no response, albeit without backlash. While other socio-demographic variables do not directly influence labeling responsiveness once political ideology is accounted for, some exert indirect effects through their relationship with ideology. Furthermore, we find that the effects of labeling among liberals persist over the medium term (four months) but not in the long-term (nine months). These findings highlight the need for a targeted approach to eco-labeling that considers ideological and socio-demographic heterogeneity.

## Figures and Tables

**Table 1:** Regression results for Study 1.

	Choice of Lower-Emission Product (Label E = 1 to A = 5)	
Independent Variable	B	SE
Labeling proportion	0.13 *	0.06
Political ideology (conservative) ^a^	−0.04	0.03
Age (older)	0.06	0.05
Gender (female)	0.18	0.10
Ethnicity (white)	−0.15	0.12
Urbanization level	−0.08	0.05
Higher education	−0.01	0.05
Higher subjective SEP	0.12 *	0.06
Executive/professional ^b^	−0.08	0.23
Farmer/artisan/shopkeeper or entrepreneur ^b^	−0.12	0.20
Middle management ^b^	0.005	0.14
Student ^b^	0.17	0.28
Occupation not answered ^b^	−0.06	0.14
Intercept	3.46 ***	0.08
Labeling proportion × Political ideology (conservative)	−0.06 *	0.03
Labeling proportion ×	−0.01	0.05
Age (older)		
Labeling proportion × Gender (female)	0.11	0.10
Labeling proportion × Ethnicity (white)	−0.02	0.12
Labeling proportion × Urbanization level	−0.002	0.05
Labeling proportion × Higher education	−0.07	0.06
Labeling proportion × Higher subjective SEP	0.02	0.05

* p < 0.05; *** *p* < 0.001. ^a^ Political ideology is centered at 4, standing for centrist. All other variables are mean-centered or binary (−½;½) variables if not stated otherwise. ^b^ Occupation is a categorical variable. The reference category is employee.

**Table 2:** Regression results for Study 2.

	Shift to Lower-Emission Products Between the First and Second Choice	
Independent Variable	B	SE
Labeling proportion	0.25 ***	0.04
Political ideology (conservative) ^a^	−0.08 ***	0.02
Age (older)	0.04	0.04
Gender (female)	0.07	0.08
Ethnicity (white)	−0.13	0.09
Urbanization level	0.03	0.04
Higher education	0.02	0.04
Higher subjective SEP	0.04	0.05
Executive/professional ^b^	0.19	0.17
Farmer/artisan/shopkeeper or entrepreneur ^b^	−0.14	0.17
Middle management ^b^	−0.0005	0.11
Student ^b^	−0.24	0.23
Occupation not answered ^b^	0.11	0.12
Misinformed condition ^c^	0.24 **	0.08
Intercept	0.62 ***	0.07
Labeling proportion × Political ideology (conservative)	−0.05 *	0.02
Labeling proportion ×	−0.06	0.04
Age (older)		
Labeling proportion × Gender (female)	0.19 *	0.08
Labeling proportion × Ethnicity (white)	0.07	0.09
Labeling proportion × Urbanization level	−0.04	0.04
Labeling proportion × Higher education	−0.008	0.05
Labeling proportion × Higher subjective SEP	0.02	0.04

* *p* < 0.05; ** *p* < 0.01; *** *p* < 0.001. ^a^ Political ideology is centered at 4, standing for centrist. All other variables are mean-centered or binary (−½;½) variables if not stated otherwise. ^b^ Occupation is a categorical variable, and the reference is employee. ^c^ This indicates the condition in which consumers were informed about the right or correct wording meaning of CO_2_ emissions linked to carbon labeling and serves as a control variable.

**Table 3:** Regression results for Study 3 (medium-term and long-term effects).

	Intentions to Purchase A or B Products				Intentions to Purchase D or E Products			
	Medium Term (Cut Fruit)		Long Term (Instant Meal)		Medium Term (Cut Fruit) ^d^		Long Term (Instant Meal)	
Independent Variable	B	SE	B	SE	B	SE	B	SE
Labeling proportion	0.01	0.03	0.02	0.04	0.01	0.04	−0.02	0.05
Political ideology (conservative) ^a^	−0.03 *	0.01	−0.06 **	0.02	0.15 ***	0.02	0.13 ***	0.02
Age (older)	−0.05	0.03	−0.08 *	0.04	0.08 *	0.04	−0.09 *	0.04
Gender (female)	0.02	0.05	0.05	0.07	−0.07	0.07	−0.16 *	0.08
Ethnicity (white)	0.01	0.05	0.01	0.08	−0.002	0.07	−0.14	0.09
Urbanization level	−0.01	0.03	0.01	0.04	−0.06	0.03	−0.04	0.04
Higher education	−0.03	0.03	−0.09 *	0.04	−0.02	0.04	−0.08	0.04
Higher subjective SEP	−0.01	0.03	0.05	0.04	−0.02	0.04	−0.04	0.05
Executive/professional ^b^	0.08	0.10	−0.05	0.16	0.06	0.13	0.36 *	0.18
Farmer/artisan/shopkeeper or entrepreneur ^b^	−0.12	0.10	0.14	0.14	0.26	0.13	0.21	0.16
Middle management ^b^	0.11	0.07	0.16	0.10	0.01	0.09	0.02	0.11
Student ^b^	−0.15	0.14	0.22	0.19	−0.08	0.18	−0.15	0.22
Occupation not answered ^b^	−0.06	0.07	0.10	0.09	0.09	0.09	−0.14	0.11
Misinformed condition ^c^	0.05	0.05			0.07	0.06		
Intercept	4.28 ***	0.04	3.92 ***	0.06	2.41 ***	0.05	2.56 ***	0.07
Labeling proportion × Political ideology	−0.03 *	0.01	0.04	0.02	0.01	0.02	0.004	0.02
Labeling proportion × Age (older)	−0.01	0.03	0.03	0.04	−0.03	0.03	0.02	0.04
Labeling proportion × Gender (female)	−0.02	0.05	−0.03	0.07	0.01	0.06	−0.04	0.08
Labeling proportion × Ethnicity (white)	0.01	0.05	0.01	0.08	0.03	0.07	−0.01	0.10
Labeling proportion × Urbanization level	−0.04	0.03	0.02	0.04	0.05	0.03	−0.03	0.04
Labeling proportion × Higher education	0.03	0.03	−0.02	0.04	0.004	0.04	−0.04	0.05
Labeling proportion × Higher subjective SEP	−0.02	0.03	−0.03	0.04	−0.02	0.04	0.01	0.04

* *p* < 0.05; ** *p* < 0.01; *** *p* < 0.001. ^a^ Political ideology is centered at 4, standing for centrist. All other variables are mean-centered or binary (−½;½) variables, if not stated otherwise. ^b^ Occupation is a categorical variable, and the reference is employee. ^c^ This indicates the condition in which consumers were informed about the right or correct wording meaning of CO_2_ emissions linked to carbon labeling and serves as a control variable. ^d^ Due to one missing observation for medium-term purchase intentions for D or E products in the cut fruit study, the sample size for this model is n = 1232.

## Data Availability

The original contributions presented in the study are included in the article/Supplementary Material, further inquiries can be directed to the corresponding author.

## Supplementary Materials

The following supporting information can be downloaded at: https://www.mdpi.com/article/10.3390/nu17081321/s1, Section S1: Attrition analyses, Study 1; Section S2: Attrition analyses, Study 2; Figure S1: Study 2: Example of stimuli for each condition in Choice 2 (Choice 1 is equivalent to Condition 1); Table S1: Regression results for Study 2.
