# Supplementary material for: Which Consumers Change Their Food Choices in Response to Carbon Footprint Labels? The Role of Political Ideology and Other Socio-Demographic Factors
Source: Nutrients. 2025 Apr 10;17(8):1321. doi: 10.3390/nu17081321 (PMC12030636; doi:10.3390/nu17081321)
Supplement: Supplementary file 1 [file nutrients-17-01321-s001.zip › nutrients-3557751-supplementary.pdf]

nutrients

MDPI

Supplementary Materials

Section S1: Attrition analyses, Study 1

We conducted an attrition check to examine the differences between respondents who only participated in the first stage of the first study and those who participated in both. The results indicate that both groups responded similarly to eco-labeling. We find no significant differences regarding the effect of labeling on product choice between them (B = −0.02, SE = 0.04, $p = 0.55$). We further find no significant differences between participants from the first (M = 4.21, SD = 0.79) and second stage (M = 4.23, SD = 0.79) regarding their environmental care ($t(1250) = -0.47$, $p = 0.64$).

Section S2: Attrition analyses, Study 2

We conducted an attrition check to examine the differences between respondents who only responded to the first stage of the first study and those who participated in both. The results indicate that both groups responded similarly to eco-labeling. We find no significant differences regarding the effect of labeling on product choice between them (B = 0.09, SE = 0.06, $p = 0.16$). We further find no significant differences between participants from the first (M = 4.11, SD = .79) and second stage (M = 4.16, SD = .81) regarding their environmental care ($t(1807) = -1.60$, $p = 0.11$).

Figure S1

Study 2: Example of stimuli for each condition in Choice 2 (Choice 1 is equivalent to Condition 1)

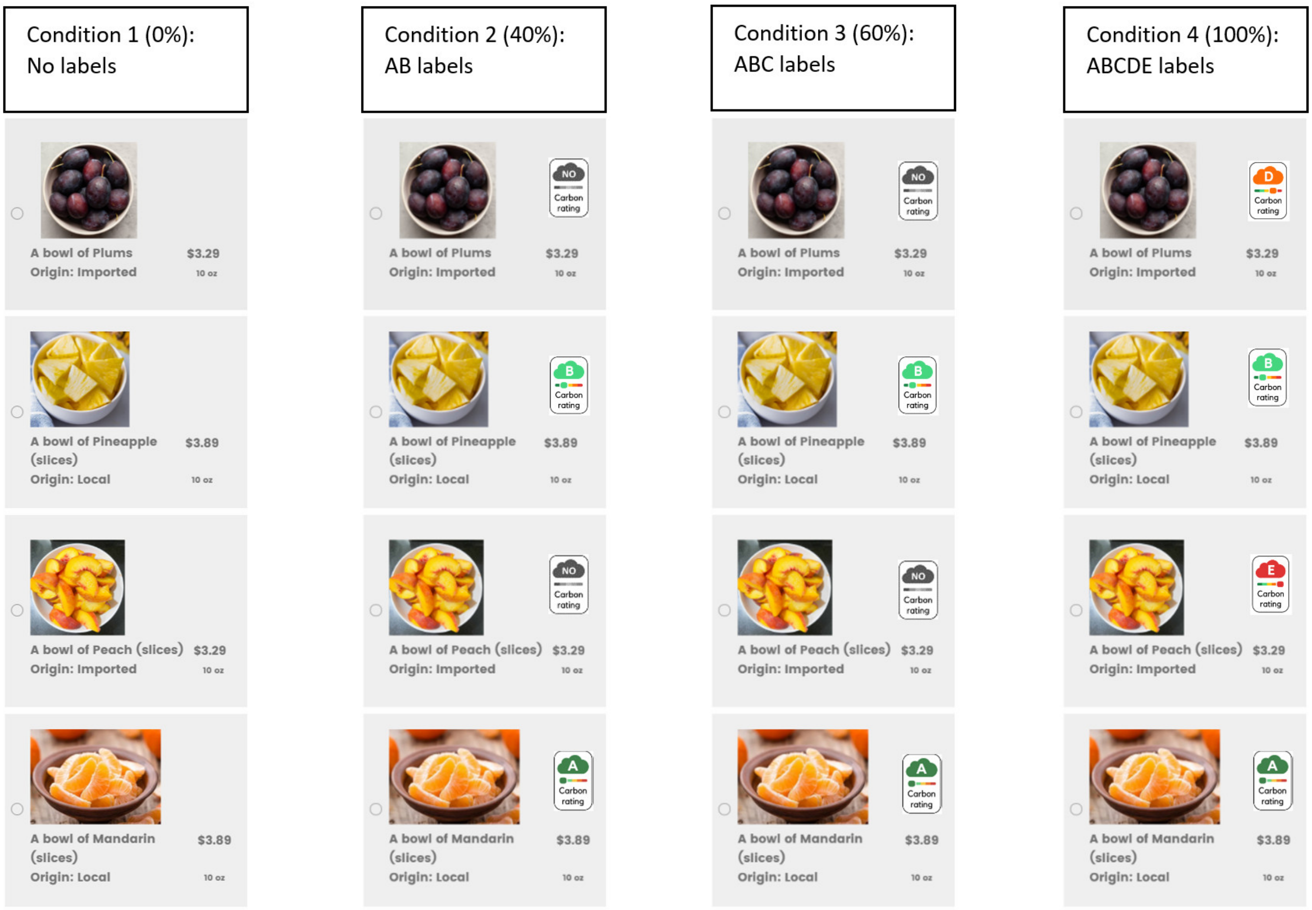

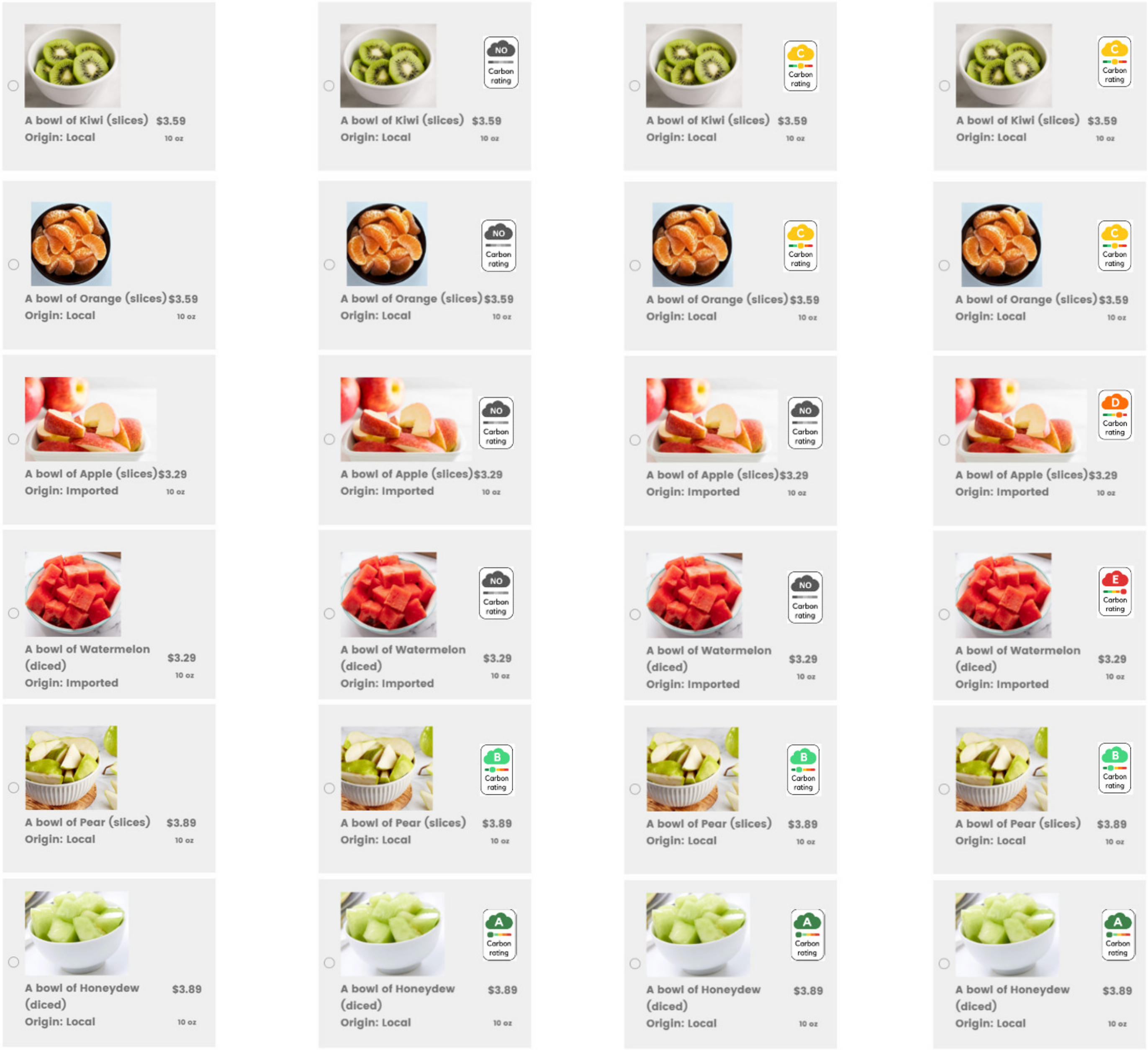
A bowl of Kiwi (slices) $3.59
Origin: Local 10 oz
NO Carbon rating
A bowl of Kiwi (slices) $3.59
Origin: Local 10 oz
C Carbon rating
A bowl of Kiwi (slices) $3.59
Origin: Local 10 oz
C Carbon rating
A bowl of Kiwi (slices) $3.59
Origin: Local 10 oz
A bowl of Orange (slices) $3.59
Origin: Local 10 oz
NO Carbon rating
A bowl of Orange (slices) $3.59
Origin: Local 10 oz
C Carbon rating
A bowl of Orange (slices) $3.59
Origin: Local 10 oz
C Carbon rating
A bowl of Orange (slices) $3.59
Origin: Local 10 oz
A bowl of Apple (slices) $3.29
Origin: Imported 10 oz
NO Carbon rating
A bowl of Apple (slices) $3.29
Origin: Imported 10 oz
NO Carbon rating
A bowl of Apple (slices) $3.29
Origin: Imported 10 oz
D Carbon rating
A bowl of Apple (slices) $3.29
Origin: Imported 10 oz
A bowl of Watermelon (diced) $3.29
Origin: Imported 10 oz
NO Carbon rating
A bowl of Watermelon (diced) $3.29
Origin: Imported 10 oz
NO Carbon rating
A bowl of Watermelon (diced) $3.29
Origin: Imported 10 oz
E Carbon rating
A bowl of Watermelon (diced) $3.29
Origin: Imported 10 oz
A bowl of Pear (slices) $3.89
Origin: Local 10 oz
B Carbon rating
A bowl of Pear (slices) $3.89
Origin: Local 10 oz
B Carbon rating
A bowl of Pear (slices) $3.89
Origin: Local 10 oz
B Carbon rating
A bowl of Pear (slices) $3.89
Origin: Local 10 oz
A bowl of Honeydew (diced) $3.89
Origin: Local 10 oz
A Carbon rating
A bowl of Honeydew (diced) $3.89
Origin: Local 10 oz
A Carbon rating
A bowl of Honeydew (diced) $3.89
Origin: Local 10 oz
A Carbon rating
A bowl of Honeydew (diced) $3.89
Origin: Local 10 oz

Table S1: Regression Results for Study 2

	Choice of lower-emission product (label E=1 to A=5) in the second choice	
**Independent variable**	**B**	**SE**
Labeling proportion	0.26***	0.05
Political ideology (Conservative) [a]	−0.11***	0.02
Age (older)	0.20***	0.05
Gender (female)	0.17*	0.09
Ethnicity (white)	−0.11	0.09
Urbanization level	0.01	0.05
Higher education	0.02	0.05
Higher subjective SEP	0.01	0.05
Executive/Professional[b]	0.31	0.17
Farmer/Artisan/Shopkeeper or Entrepreneur[b]	−0.11	0.17
Middle management[b]	0.16	0.11
Student[b]	0.19	0.25
Occupation not answered[b]	0.02	0.13
Misinformed condition[c]	−0.53***	0.08
Intercept	2.47***	0.07
Labeling proportion × Political ideology (Conservative)	−0.07**	0.02
Labeling proportion × Age (older)	−0.02	0.04
Labeling proportion × Gender (female)	0.20*	0.09
Labeling proportion × Ethnicity (white)	0.08	0.09
Labeling proportion × Urbanization level	−0.05	0.04
Labeling proportion × Higher education	0.008	0.05
Labeling proportion × Higher subjective SEP	0.03	0.05

$*p < 0.05$; $**p < 0.01$; $***p < 0.001$. [a] Political ideology is centered at 4, standing for centrist. All other variables are mean-centered or binary (−½;½) variables if not stated otherwise. [b] Occupation is a categorical variable. The reference category is employee. [c] This indicates the condition in which consumers were informed about the right or correct wording meaning of CO_2 emissions linked to carbon labeling and serves as a control variable.
